# Chronic oxidative stress adaptation in head and neck cancer cells generates slow-cyclers with decreased tumour growth in vivo

**DOI:** 10.1038/s41416-023-02343-6

**Published:** 2023-07-17

**Authors:** Julia Berner, Lea Miebach, Marcel Kordt, Christian Seebauer, Anke Schmidt, Michael Lalk, Brigitte Vollmar, Hans-Robert Metelmann, Sander Bekeschus

**Affiliations:** 1 Department of Oral, Maxillofacial, and Plastic Surgery, Greifswald University Medical Center, Ferdinand-Sauerbruch-Str, 17475 Greifswald, Germany; 2 ZIK plasmatis, Leibniz Institute for Plasma Science and Technology (INP), Felix-Hausdorff-Str. 2, 17489 Greifswald, Germany; 3 Department of General, Visceral, Thoracic, and Vascular Surgery, Greifswald University Medical Center, Ferdinand-Sauerbruch-Str, 17475 Greifswald, Germany; 4 Rudolf-Zenker-Institute of Experimental Surgery, Rostock University Medical Center, Schillingallee 69a, 18057 Rostock, Germany; 5 Institute for Biochemistry, University of Greifswald, Felix-Hausdorff-Str. 4, 17489 Greifswald, Germany; 6 Clinic and Policlinic for Dermatology and Venerology, Rostock University Medical Center, Strempelstr. 13, 18057 Rostock, Germany

**Keywords:** Oral cancer, Nanomedicine

## Abstract

**Background:**

Reactive oxygen species (ROS) are implicated in cancer therapy and as drivers of microenvironmental tumour cell adaptations. Medical gas plasma is a multi-ROS generating technology that has been shown effective for palliative tumour control in head and neck cancer (HNC) patients before tumour cells adapted to the oxidative stress and growth regressed fatally.

**Methods:**

In a bedside-to-bench approach, we sought to explore the oxidative stress adaptation in two human squamous cell carcinoma cell lines. Gas plasma was utilised as a putative therapeutic agent and chronic oxidative stress inducer.

**Results:**

Cellular responses of single and multiple treated cells were compared regarding sensitivity, cellular senescence, redox state and cytokine release. Whole transcriptome analysis revealed a strong correlation of cancer cell adaption with increased interleukin 1 receptor type 2 (IL1R2) expression. Using magnetic resonance imaging, tumour growth and gas plasma treatment responses of wild-type (WT) and repeatedly exposed (RE) A431 cells were further investigated in a xenograft model in vivo. RE cells generated significantly smaller tumours with suppressed inflammatory secretion profiles and increased epidermal growth factor receptor (EGFR) activity showing significantly lower gas plasma sensitivity until day 8.

**Conclusions:**

Clinically, combination treatments together with cetuximab, an EGFR inhibitor, may overcome acquired oxidative stress resistance in HNC.

## Introduction

Head and neck squamous cell carcinoma (HNSCC) is the sixth most common malignancy worldwide, accounting for more than 800,000 new cases annually [1]. Besides inherited genes predisposing individuals to this specific tumour entity, oncogenesis is mainly driven by constant exposure to carcinogens like heavy tobacco use and alcohol consumption [2]. Also, the pre-infection with high-risk types of human papillomavirus (HPV) is considered to put patients at risk for developing HNSCC [3]. Despite improvements in diagnosis and implementation of novel therapeutic approaches in recent decades, the prognosis of patients suffering from HNSCC remains poor, with a five-year overall survival rate below 50% [1, 4]. Severe side effects of conventional treatment strategies and multidrug resistance of cancer cells are a major obstacle for attending clinicians, limiting therapeutic options and leading to tumour relapse even in aggressive combination strategies [5]. To improve outcomes and the life quality of patients, novel treatment strategies are urgently needed [6].

Medical gas plasma technology has become of interest in dermato-oncological research in recent years. Similar to other reactive oxygen species (ROS) and nitrogen species (RNS)-generating therapies already applied in clinical oncology, e.g., photodynamic therapy, this innovative treatment modality elicits its anti-tumour activity via targeting the tumour redox state [7]. Preclinical in vitro and in vivo studies are promising [8–13], and beneficial results have already been reported for patients suffering from therapy refractory head and neck cancers [14–17]. Gas plasmas are unique in their ability to generate a multitude of highly reactive ROS/RNS simultaneously, causing irreversible damage to intracellular biomolecules and, consequently, induction of cell death signalling pathways [18–20]. Despite direct tumour toxic effects, plasma-derived ROS have an additional advantage in HNSCC. Due to their antimicrobial efficacy, they can decrease microbial growth on advanced-stage tumours, where tissue contamination is accompanied by hostile odour that negatively affects social interactions and the patient’s quality of life [15]. However, tumour relapse due to acquired resistance of initially responsive patients can be a major challenge in some cases. The need to shed light on mechanisms underlying the adaption of tumours to medical gas plasma therapy is underlined by a clinical case where partial tumour remission was observed in a 54-year-old male patient who underwent palliative intended gas plasma therapy after being diagnosed with an exulcerating, superinfected squamous cell carcinoma of the oral floor. Unfortunately, while still under gas plasma therapy, the patient showed a progressive disease eight months later and passed away shortly after (Supplementary Fig. S1).

In this light, we established a model system for repeated gas plasma exposure in two squamous cell carcinoma cell lines in vitro. A431 and SCC-25 were exposed to gas plasma weekly in eight treatment cycles. Cellular responses were compared in wild-type (WT) and repeatedly exposed (RE) cancer cells with regard to toxicity, intracellular ROS levels, cellular senescence, and release of twenty different cytokines and growth factors. Whole transcriptome gene expression analysis served to identify differentially expressed genes that correlated with adaption to gas plasma-induced toxicity. Treatment responses of WT and RE A431 cells were further investigated in a xenograft model of HNSCC in vivo.

Overall, the present study is the first to comprehensively investigate the adaption processes of malignant cells to repeated oxidative stress conditions induced by medical gas plasmas and might help to identify potential targets to overcome resistance in such cases.

## Materials and methods

### Gas plasma jet and treatment

For palliative tumour treatment in the patient, the plasma jet kINPen MED (neoplas, Germany), an accredited medical device class IIa in Europe and routinely applied in clinical dermatology, was utilised [21]. The jet contains a grounded outer electrode and works with a pin-type powered electrode inside a 1.6 mm-thin (inner diameter) dielectric ceramic tube. Plasma was ignited at a frequency of 1 MHz using 5 slm of argon feed gas. The operating distance from the pencil to the skin surface was approximately 1 cm. Therapeutically application of gas plasma was performed for 5 min in cycles of 2–3 single treatments within 1 week. The atmospheric pressure plasma jet kINPen (neoplas) was used for experimental gas plasma treatments. The device is technically similar to the kINPen MED, and its physicochemical properties have been extensively described before [22]. The jet was operated with argon (purity 99.9999%; Air Liquide, France) at either 1.5 (in vitro) or 5 (in vivo) standard liters per minute (slm) excited at the electrode within the head of the kINPen at a frequency power of 1 MHz and a generating power of about 1 W. Using a computer-controlled and motorised xyz table (CNC, Germany), the kINPen hovered over the centre of each well at a distance of 1 cm between nozzle tip and liquid surface in in vitro treatments. The evaporated liquid was supplemented by adding predetermined amounts of double-distilled water immediately after gas plasma treatment. The operating distance from nozzle to the skin surface was about 1 cm in animal experiments.

### Quantification of reactive oxygen and nitrogen species

Profiling of reactive oxygen (ROS) and nitrogen species (RNS) fingerprints in liquids was done immediately after plasma treatment without or in the presence of wild-type A431 and SCC-25 cells. Briefly, 100k cells were seeded in 750 µl PBS per well of a 24 flat bottom plate and exposed to plasma for 15 s. Relative assessment of short-lived species was done using the redox-sensitive, fluorescent probes aminophenyl fluoresceine (APF) and hydroxyphenyl fluoresceine (HPF; both Enzo Life Sciences, Germany) sensitive to detecting hydroxyl radicals (^.^OH), peroxynitrite (ONOO–; both APF and HPF) and hypochlorous acid (HOCl; APF only). A singlet oxygen sensor (SOS; Thermo Fisher Scientific, Germany) was used for detection of singlet oxygen (^1^O_2_) and diaminofluoresceine (DAF; Thermo Fisher Scientific) for the detection of nitric oxide (NO^.^). Briefly, 90 µl of plasma-treated samples were added to 10 µl of a 10 µM sensor solution to reach a final concentration of 1 µM in a 96-well flat bottom plate (Sarstedt, Germany). Immediately after, fluorescence was determined at *λ*_ex_ 485 nm and *λ*_em_ 525 nm using a multiplate reader (F200; Tecan, Switzerland). Quantification of hydrogen peroxide (H_2_O_2_) deposition was done using the *Amplex Ultra Red* Assay (Thermo Fisher Scientific) according to the supplier’s instructions. Fluorescence was assessed at *λ*_ex_ 535 nm and *λ*_em_ 590 nm using a multiplate reader (F200; Tecan). Absolute concentrations were calculated against a standard curve. For lifetime measurements of H_2_O_2,_ treatment and liquid analysis of cell-free cell culture medium was performed as described before, and H_2_O_2_ quantification was performed immediately, 1 h, 2 h, 4 h, 8 h, 12 h and 24 h after plasma treatment.

### Long-term cell culture

The human epithelial squamous cell carcinoma cell lines SCC-25 and A431 (both ATCC, USA) were cultured in Roswell Park Memorial Institute (RPMI) 1640 medium (Pan Biotech, Germany) supplemented with 10% foetal bovine serum, 1% penicillin, 1% streptomycin and 1% glutamine (all Corning, Germany). Cells were kept under standard culture conditions at 37 °C, 95% humidity, and 5% CO_2_ in a cell culture incubator (Binder, Germany). To establish an in vitro model of repeated gas plasma treatment, cells were exposed to gas plasma weekly in eight treatment cycles. Therefore, 1 × 10^5^ cells in 750 µl fully supplemented medium were seeded per well in a 24-well plate (Greiner Bio-One, Germany) and treated with gas plasma immediately. After 24 h of incubation, cells were harvested, transferred to a new cell culture flask, and cultivated until the treatment cycle of the following week. Cells and culture supernatants were collected for downstream analysis after single exposure and multiple treatment cycles for downstream analysis. Wild-type (WT) and repeatedly exposed (RE) cells after eight treatment cycles were cryopreserved for in vivo experiments. Due to the experimental complexity, as well as time intensive and elaborate study design, the long-term cell culture experiment was performed once for each cell line, representing one single biological replicate.

### Cellular morphology

High-content imaging (Operetta CLS; PerkinElmer, Germany) was performed 24 h after gas plasma exposure to monitor morphologic alterations after single and multiple treatment cycles. Brightfield images were acquired using a ×20 Air objective (NA 0.4; Zeiss, Germany) in 132 fields of view. The experimental setup was done using Harmony 4.9 software (PerkinElmer).

### Dose finding

To ensure culture regrowth after plasma exposure until the subsequent treatment cycle, the IC25 value was predetermined to achieve low levels of cytotoxicity upon repeated plasma exposure. The metabolic activity of cancer cells and the human non-malignant HaCaT keratinocyte cell line (ATCC, USA) was evaluated using the Alamar blue assay. Therefore, 100 µM of 7 hydroxy-3H-phenoxazin-3-on-10-oxid (resazurin; Alfa Aesar, USA) was added to the cells 20 h after gas plasma exposure, followed by incubation for 4 h. Viable cells metabolise non-fluorescent resazurin to fluorescent resorufin in a NADH/H+-dependent reaction, thereby indicating the cells' metabolic state [23]. Fluorescence intensities were assessed at *λ*_ex_ 535 nm and *λ*_em_ 590 nm using a microplate reader (F200; Tecan). Resazurin without cells was used for background subtraction. Data were normalised to untreated controls.

### Proliferation assay and cellular viability

The WST-1 proliferation assay was conducted to analyse the proliferation of cancer cells following gas plasma exposure. Therefore, 75 µl of the WST-1 Premix solution (Takara Bio, Japan) was added to each well 22 h after treatment. Following incubation for 2 h at 37 °C and 5% CO_2_, the absorbance was measured at 440 nm using a microplate reader (M200; Tecan). Cell-free medium was used for background subtraction. Data were normalised to untreated controls. Cellular viability was further assessed 24 h after gas plasma treatment using flow cytometry. Briefly, cells were stained with 1 µM iFluor 840 maleimide (AAT Bioquest, USA; Cat# 1402) for 20 min at 37 °C. After washing, cells were acquired using flow cytometry (CytoFLEX LX; Beckman-Coulter, Germany) and evaluated using Kaluza 2.1.3 analysis software (Beckman-Coulter).

### Cell cycle analysis and intracellular ROS levels

Cell cycle analysis and assessment of intracellular ROS levels was performed on fixed and permeabilized cells 24 h after gas plasma treatment. Nucleic staining was done using 10 µM 4′,6 diamidine-2-phenylindole (DAPI; Sigma‑Aldrich, Germany), and a PE-conjugated antibody was utilised to stain 3-nitrotyrosine (Santa Cruz Biotechnology, USA; Cat# sc-32757 PE) for 20 min at 37 °C. After washing, cells were acquired using flow cytometry (CytoFLEX LX; Beckman-Coulter) and evaluated using Kaluza 2.1.3 analysis software (Beckman-Coulter).

### GSH/GSSG assay

The GSH/GSSG-Glo assay (Promega, Germany) was used to determine the intracellular GSH/GSSG ratio. Briefly, cells were exposed to plasma as described before. Immediately after, 150 µl cell suspension of each well was transferred in a white 96-well flat bottom plate (Nunclon; Thermo Fisher Scientific). Following incubation for 24 h, the medium was aspirated, and cells were lysed with total or oxidised glutathione reagent. Cells were incubated with luciferin generation reagent for 30 min at room temperature before adding the luciferin detection reagent. Luminescence was measured using a multimode plate reader (M200; Tecan), and GSH/GSSG concentrations were calculated against a standard curve.

### Western blot

Cells were collected 6 h and 24 h after plasma exposure, and pellets were lysed in 100 µl RIPA buffer containing protease and phosphatase inhibitors (cOmplete Mini, phoSTOP, PMSF; Sigma-Aldrich). Sonication was performed to disrupt cellular membranes. Protein expression levels of catalase (Cat; Santa Cruz Biotechnology, USA; Cat# sc-271803) and superoxide dismutase 1 (SOD1; Invitrogen, USA; Cat# PA5-27240) were assessed using appropriate monoclonal antibodies and capillary-based gel electrophoresis performed using the WES system (ProteinSimple, Germany) according to the manufacturer’s protocol. Band intensities were quantified using *Compass for Simple Western* Software and normalised to housekeeping control GAPDH expression.

### RNA isolation and gene expression profiling

RNA was isolated from cell pellets using an RNA isolation kit (Bio & Sell, Germany) according to the manufacturer’s protocol. Prior gene expression analysis using a single-colour microarray kit (Agilent Technologies, Germany), RNA concentrations of each sample were determined using a spectrophotometer (NanoDrop 2000C; Thermo Fisher Scientific). According to the manufacturer’s instructions, 200 ng of sample RNA was used for complementary DNA (cDNA) synthesis and amplification following transcription into complementary RNA (cRNA). Purification of 600 ng cyanine-3-labelled cRNA was followed by microarray hybridisation at 65 °C for 17 h. After washing (Gene Expression Wash Buffer Kit; Agilent Technologies, Germany), slides were dried and scanned immediately (Agilent SureScan; Agilent Technologies) using a 61 ×21.6 mm scan area, with a 3 µm resolution at 20 bit. Data were extracted with the Agilent Feature Extraction Software 10.7.3 and evaluated using the GeneSpring software 14.9.1 (both Agilent Technologies). The housekeeping *ACTB* was used as a reference control. Gene expression of single and multiple gas plasma-treated tumour cells was compared to wild-type controls. Annotated genes with significantly different expression (*P* ≤ 0.05) and a fold change ≤5 were classified by Gene Ontology (GO) pathway analysis using PANTHER database networks. Microarray data were deposited into the gene expression omnibus database (GSE223042).

### Animal experiments

Experimental procedures were reviewed and approved by the local authority *Landesamt für Landwirtschaft, Lebensmittelsicherheit und Fischerei* (LALLF) and the ethical committee of Mecklenburg-Vorpommern (approval number: 7221.3-1-057/18). Group size was chosen (*n* = 8 per group) based on sample size calculation (alpha = 0.05, beta = 0.20, power = 0.8) prior to experiments. Animals were allocated to one of the four groups using randomly generated numbers (Random number generator, Stat Trek, https://stattrek.com/statistics/random-number-generator.aspx). Six hundred thousand wild-type (WT) or repeatedly gas plasma-exposed (RE) A431 cells in 1:1 PBS/Matrigel (Corning) were injected subcutaneously in both flanks of male NOD.Cg-Prkdc^scid^ Il2rg^tm1Wjl^/SzJ (NSG) mice (JAX mice; Charles River, Germany) aged 8–20 weeks. These mice developed an immunodeficiency due to mutations of the DNA repair complex protein Prkdc (protein kinase, DNA-activated, catalytic polypeptide; scid mutation; severe combined immune deficiency) and a complete null allele of the interleukin (IL) 2 receptor common gamma chain (IL2rg^null^) to permit engraftment of human tumour cells in a xenograft in vivo model. Mice were bred under specific germ-free conditions in individually ventilated cages. Starting day four after injection, tumours on both flanks were exposed to gas plasma every four days. Tumour growth was monitored using daily caliper measurements. Animals were sacrificed on day 24.

### Magnetic resonance imaging

Magnetic resonance imaging (MRI) was performed 22 days post tumour cell injection using a 7 Tesla small animal MRI scanner (BioSpec 70/30; Bruker BioSpin MRI, Germany) with a 1 H transmit resonator and a 2-by-2 receive-only surface coil array positioned on the back of the mice. Tumour size was assessed based on high-resolution T2-weighted TurboRARE imaging sequences in the transversal plane. T2-weighted sequences were acquired according to the following parameters: 4.200 ms repetition time, 26.0 ms echo time, 42 mm × 24 mm field of views, 351 × 200 matrix, 0.12 × 0.12 × 0.75 mm^3^ voxel size, 35–50 slices depending on tumour size and a total acquisition time of approx. 10 min. Tumour volume was analysed using ITK-SNAP 3.6.0 software (Penn Image Computing and Science Laboratory, USA).

### Tumour digestion and flow cytometric analysis

Excised tumours were digested using a tumour dissociation kit and tumour dissociator (OctaMACS; both Miltenyi Biotech, Germany) to create single-cell suspensions for flow cytometric analysis of intra- and extracellular marker expression. Cells were fixed immediately after digestion using 4% paraformaldehyde. Surface marker expression staining was done using monoclonal antibodies (Table 1) targeted against (conjugate) HLA-ABC (AF700), CD152 (BV785), CD274 (BV650), Fas (BV510), EGFR (APC/Fire750), CD155 (PerCP/Cy5.5), EpCam (BV605; all BioLegend, The Netherlands), calreticulin (CRT; PE; Enzo Life Sciences), heat shock protein (HSP) 70 (APC; Novus Biologicals) and murine HLA-ABC (BUV661; BD Biosciences). Intracellular staining was done after cell permeabilization (Intracellular Staining Permeabilization Wash Buffer; BioLegend) using monoclonal antibodies targeted against (conjugate) 8-OhdG (AF488; Santa Cruz Biotechnology, USA; Cat # sc-393871 AF488), EGFR ~ P (AF647; Abcam; Cat# ab205828), γH2AX (APC/Fire750; BioLegend; Cat # 613422), Ki-67 (BV605; BioLegend; Cat # 350522) and 3-nitrotyrosine (PE; Santa Cruz Biotechnology, USA; Cat # sc-32757 PE). After incubation for 1 h at 4 °C, cells were washed and acquired by flow cytometry (CytoFLEX LX). Data analysis was performed using Kaluza 2.1.3 analysis software (Beckman-Coulter) and Spotfire 7.9.1 (TIPCO, USA).

**Table 1:** Monoclonal antibodies used for cell surface staining.

Ligand	Fluorochrome	Clone	Supplier	Cat #
CD40	FITC	5C3	BioLegend	334306
CD45	PE/Cy7	2D1	BioLegend	368532
CD80	PE/Dazzle	2D10	BioLegend	305230
CD152	BV785	BNI3	BioLegend	369624
CD155	PerCP/Cy5.5	SKII.4	BioLegend	337612
CRT	PE	FMC75	Enzo Life Sciences	ADI-SPA-601PE-F
EGFR	APC/Fire750	AY13	BioLegend	352926
EpCam	BV605	9C4	BioLegend	324224
Fas	BV510	DX2	BioLegend	305640
HSP70	APC	N27F3-4	Novus Biologicals	NB110-96425APC
MHC1	AF700	W6/32	BioLegend	311438
MHC1	BUV661	M1/42	BD Biosciences	749702
PD-L1	BV650	29E2A3	BioLegend	329740

*AF* Alexa Fluor, *APC* allophycocyanin, *BV* brilliant violet, *BUV* brilliant ultraviolet, *Cy* cyanin, *FITC* fluorescein isothiocyanate, *PE* phycoerythrin, *PerCP* peridinin-chlorophyll-protein.

### Chemokine and cytokine quantification

Chemokine and cytokine analysis were performed on in vitro cell culture supernatants collected 24 h after gas plasma treatment from three technical replicates and ex vivo collected tumour supernatants after tumour digestion (*n* = 8) using bead-based sandwich multianalyte assays (BioLegend) according to the supplier’s instructions. The experiment was carried out with four technical replicates per biological replicate. The assay panels consisted of beads targeted against a) interferon (IFN) α2, IFNγ, IL1β, IL6, IL8, IL10, IL12p70, IL17A, IL18, IL23, IL33, monocyte chemoattractant protein (MCP) 1, tumour necrosis factor (TNF) α or b) angiopoietin (ANGPT) 2, granulocyte colony-stimulating factor (G-CSF), granulocyte-macrophage colony-stimulating factor (GM-CSF), macrophage colony-stimulating factor (M-CSF), platelet-derived growth factor (PDGF) AA, stem cell factor (SCF), tumour growth factor (TGF) α and vascular endothelial growth factor (VEGF). After washing, beads were labelled with fluorescent detection antibodies and acquired using flow cytometry (CytoFLEX S). Absolute concentrations were calculated against standard curves using LEGENDplex 8.0 software (Vigene Tech, USA).

### Statistical analysis

Statistical analysis and graphing were performed using Prism 9.5.1 (GraphPad Software, USA) and *t* test or one- or two-way analysis of variances (ANOVA) as stated in the figure legends. Data show mean ± standard error of the mean (SEM) if not indicated otherwise. The number of technical replicates is given in the corresponding method section or figure legend. Levels of significance were as follows: ns = non-significant, *P ≤ 0.05, **P ≤ 0.01, ***P ≤ 0.001. Outliers among technical replicates were excluded using the *Identify outliers* function (ROUT, Q = 1%) in Prism 9.5.1.

## Results

### Clinical case: acquired resistance to medical gas plasma therapy impairs proceeding tumour remission in a 54-year-old HNSCC patient

In March 2015, a 54-year-old male patient was diagnosed with an ulcerating squamous cell carcinoma localised at the lateral floor of the mouth. After radical tumour resection, the tumour was classified at stage II (TNM: pT2 pN0 pM0 pL0 pV0 G2), followed by several attempts to restrict tumour progression, including radiotherapy, cisplatin, and 5-fluorouracil-based chemotherapy. Despite aggressive, multimodal approaches, tumour growth proceeded over the anterior base of the mouth with ulceration and submental tumour breakthrough (Supplementary Fig. S1a). Due to the patient’s weak general condition, a supportive palliative cancer treatment using medical gas plasma was started in October 2016. The ulcerated and bacterially superinfected tumour area was treated with the kINPen MED, which is approved as a medical device class IIa in Europe. Treatments were carried out within a distance of app. 10 mm for 5 min every two to three days. Macroscopic and microbiological examination revealed a reduction of bacterial colonisation associated with decreased wound odour. Moreover, partial remission of superficial tumour mass was observed (Supplementary Fig. S1b**)**. However, tumour progression occurred eight months later, despite continued gas plasma therapy (Supplementary Fig. S1c). The patient succumbed to his disease four months later.

### Human squamous cell carcinoma cells adapt to repeated gas plasma exposure in vitro

**Fig. 1:**
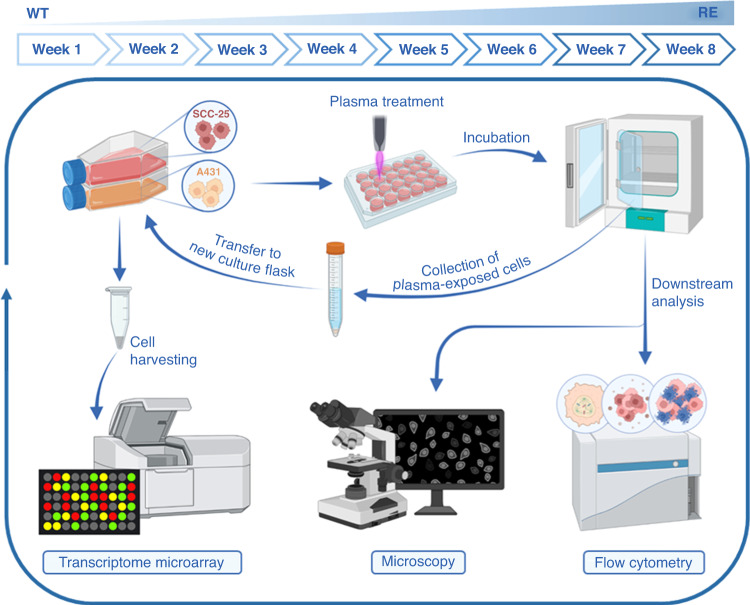
Schematic overview of the established long-term cell culture. Human SCC-25 and A431 squamous cell carcinoma cells were exposed weekly to gas plasma over 8 weeks. SCC-25 and A431 cells were seeded in 24-well plates and immediately exposed to gas plasma. After 24 h of incubation, cells were harvested and transferred to a new culture flask. Cells and culture supernatants were collected for downstream analysis 24 h or 1 week post-exposure. RE repeatedly exposed, WT wild type.

**Fig. 2:**
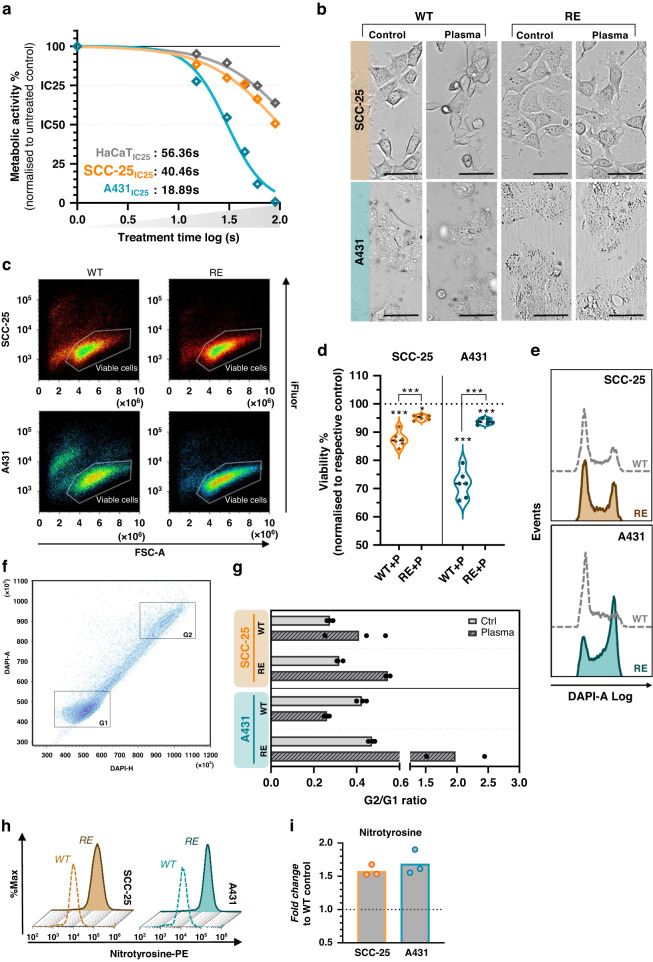
Human squamous cell carcinoma cells adapt to repeated gas plasma exposure in vitro. **a** Metabolic activity of malignant squamous cell carcinoma cells (A431, SCC-25) and non-malignant HaCaT keratinocytes 24 h post plasma treatment and calculated IC25 values (*n* = 2, except HaCaT (*n* = 5)). **b**  Representative brightfield images of cancer cells after one single or multiple treatment cycles (*n* = 3). **c**, **d** Representative flow cytometry dot plots of cells (**c**) and quantification of viable SCC-25 and A431 cells after one single or multiple treatment cycles (*n* = 8), violin plots show median (indicated as stacked line), quartiles (indicated as dotted line) and individual data points, statistical analysis was performed using two-way analysis of variance (ANOVA) with Tukey’s post hoc testing (**P ≤ 0.01, ***P ≤ 0.001) (**d**). **e** Representative flow cytometry intensity histograms of DAPI for cell cycle analysis. **f** Representative flow cytometry dot plot of DAPI+ cells showing cells in G1 and G2 phase. **g** G2/G1 phase ratio in wild-type or repeatedly exposed SCC-25 and A431 cells 24 h after gas plasma treatment (*n* = 3), bar graphs show mean and individual data points. **h**, **i** Representative flow cytometry intensity histograms of nitrotyrosine levels in wild-type or repeatedly exposed SCC-25 and A431 cells (**h**) and quantification thereof showing the baseline fold change normalised to untreated wild-type cells (*n* = 3), bar graphs show mean and individual values (**i**). Scale bar = 50 µm.

Medical gas plasma technology has successfully been shown to reduce tumour burden in patients suffering from advanced head and neck carcinoma, but acquired therapy resistance can be a major challenge, as illustrated previously (Supplementary Fig. S1). To investigate underlying molecular mechanisms in vitro, a standardised model system of repeated gas plasma exposure was established. Therefore, human squamous cell carcinoma cells SCC-25 and A431 were exposed to medical gas plasma weekly for a total of eight treatment cycles. Cells and culture supernatants were collected after gas plasma treatment of wild-type (WT) and repeatedly exposed (RE) cells for downstream analysis (Fig. 1). The calculated inhibitory concentration 25 (IC_25_) of A431 cells, showing an augmented sensitivity compared to SCC-25, was predefined as final exposure time for subsequent experiments (Fig. 2a). Human non-malignant HaCaT keratinocytes were less affected under those treatment conditions as their calculated IC25 was higher compared to their malignant counterparts pointing to a partial selectivity of plasma. In this setting, plasma treatment yielded deposition of 40 µM hydrogen peroxide (H_2_O_2_; Supplementary Fig. S2a), remaining stable over 24 h (Supplementary Fig. S2b). Moreover, a relative increase in short-lived species, including hydroxyl radicals (^.^OH), peroxynitrite (ONOO^−^; Supplementary Fig. S2c), singlet oxygen (^1^O_2_; Supplementary Fig. S2d), and nitric oxide (NO^.^; Supplementary Fig. S2e), was observed. Interestingly, relative and absolute levels of ROS/RNS were found to be diminished in the presence of cells (Supplementary Fig. S2). Twenty-four hours after a single treatment, morphological alterations indicative of terminal cell death were observed in both A431 and SCC-25 cells. Cellular rounding was not noticed after multiple treatment cycles, while cells appeared enlarged and less structured in control and gas plasma-treated cells after repeated exposure (Fig. 2b). Lack of proliferation impairment 24 h after plasma treatment indicated a significantly diminished sensitivity of SCC-25 and A431 cells towards gas plasma after multiple treatment cycles (Supplementary Fig. S3a). Resistance establishment was further confirmed by flow cytometric analysis (Supplementary Fig. S3b, c) of cell count indicative of less reduced proliferation (Supplementary Fig. S3d) and cellular viability (Supplementary Fig. S3e) using two separate methods (Fig. 2c) validating gas plasma insusceptibility of RE cells (Fig. 2d). Cell cycle alterations are a hallmark of slow-cycling persister cells and have been linked to resistance towards ROS recently. In this regard, we investigated the distribution of wild-type and repeatedly exposed cells in G1 and G2 phases of the cell cycle 24 h after gas plasma treatment (Fig. 2e). Flow cytometric analysis of G2/G1 ratios (Fig. 2f) showed a strikingly increased G2 arrest in multiple gas plasma-challenged A431 cells (Fig. 2g). Gas plasma mediated toxicity is suggested to be ROS/RNS dependent. Short-lived species might interact to form secondary agents that are able to modify and damage cellular proteins irreversibly, resulting in terminal cell death signalling. Assessment of tyrosine nitration of intracellular biomolecules is a benchmark test to monitor oxidative stress in cells. Interestingly, flow cytometric analysis of 3-nitrotyrosine residues in repeatedly exposed cancer cells (Fig. 2h) displayed a baseline increase compared to wild-type cells in A431 and SCC-25 cells (Fig. 2i). Along similar lines, repeatedly exposed A431 cells exhibited elevated basal levels of oxidised GSH (GSSG) but were able to maintain a stable GSH/GSSG ratio upon plasma treatment (Supplementary Fig. S4a). Transient adaption to oxidative stress often involves the de novo synthesis of enzymes involved in antioxidant defence. Evaluation of catalase and superoxide dismutase (SOD) 1 expression by Western blot (Supplementary Fig. S4b) showed increased baseline expression levels of catalase (Supplementary Fig. S4c) and SOD1 in cells after repeated plasma treatment. Interestingly, SOD1 expression was diminished 6 h after plasma treatment in both WT and RE cells but recovered after 24 h (Supplementary Fig. S4d).

### Repeated gas plasma exposure alters cytokine profiles released by squamous cell carcinoma cells

**Fig. 3:**
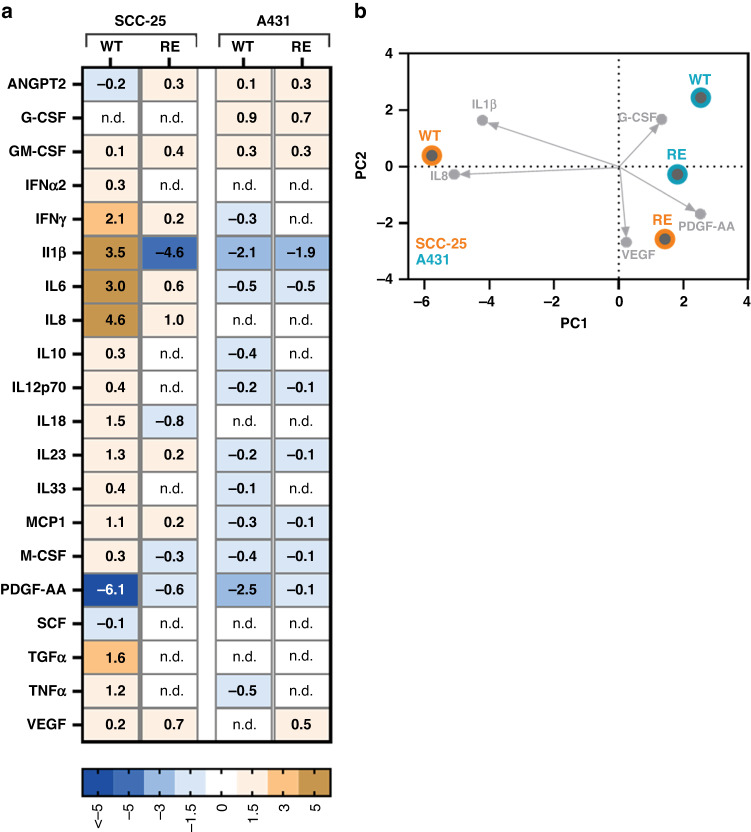
Repeated gas plasma exposure alters cytokine profiles released by squamous cell carcinoma cells. **a** Heatmap showing median cytokine levels in SCC-25 and A431 cells 24 h after gas plasma treatment in wild-type or repeatedly exposed cells normalised to respective controls. **b** Principal component analysis (PCA) calculated from normalised secretion profiles of wild-type or repeatedly exposed SCC-25 and A431 cells.

Tumour cell fate is governed by intrinsic cell properties closely engaged with the external influence of the surrounding tumour microenvironment. In a bidirectional interaction, cancers are moreover able to shape their environment to design a setting that enables optimal growth conditions. In this light, release of twenty cytokines and growth factors was quantified in culture supernatants of SCC-25 and A431 WT and RE cells collected 24 h after gas plasma treatment. Absolute levels were normalised against respective untreated controls to compare alterations in secretion profiles in response to gas plasma treatment after multiple treatment cycles. After a single exposure, elevated levels of interferon (IFN) y, interleukin (IL) 1, IL6, IL8, IL18, IL23, monocyte chemotactic protein (MCP) 1, tumour growth factor (TGF) α, and tumour necrosis factor (TNF) α paralleled by a reduction in platelet-derived growth factor (PDGF) AA were found in SCC-25 WT cells. Alterations in IL1β and PDGF-AA release were also found in A431 WT cells, but remarkable differences were overall less prominent compared to SCC-25 cells. After multiple gas plasma treatment cycles, RE cells displayed distinct cytokine profiles striking for mitogenic PDGF-AA in both cell lines. (Fig. 3a). Principal component analysis calculated from relative cytokine levels underlined the differences between wild-type and repeatedly exposed cells (Fig. 3b).

### Repeated gas plasma exposure alters gene expression profiles of squamous cell carcinoma cells

**Fig. 4:**
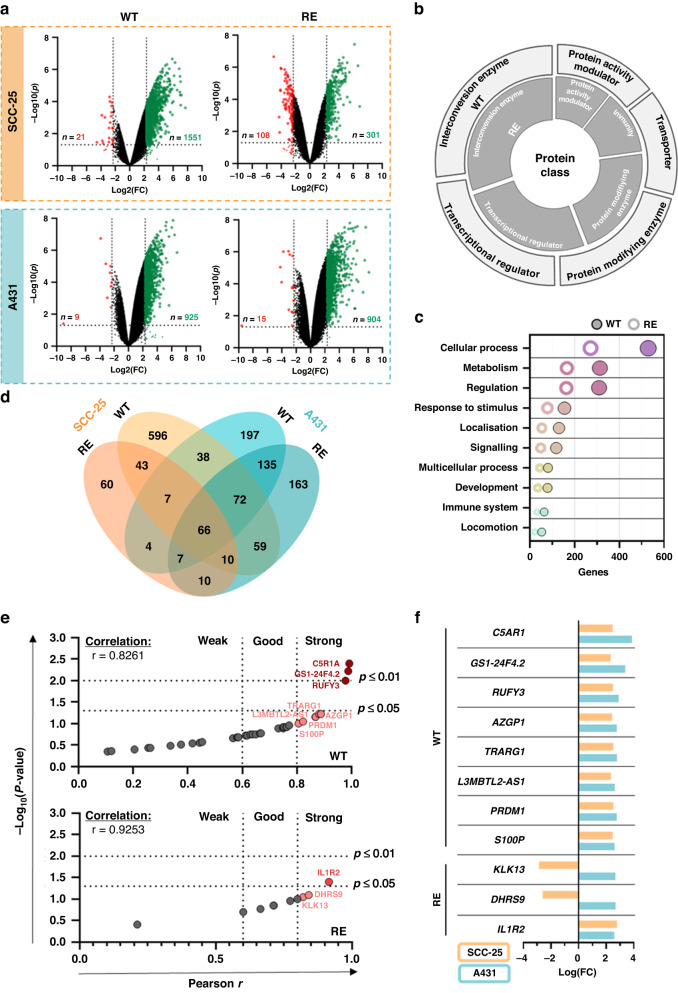
Repeated gas plasma exposure alters gene expression profiles in SCC-25 and A431 squamous cell carcinoma cells. **a** Volcano plot showing significantly up- (green) and downregulated (red) genes after single (*n* = 4) or repeated gas plasma exposure (*n* = 4) compared to untreated wild-type (*n* = 2) SCC-25 and A431 cells, statistical analysis was performed using moderate *t* test (two-tailed). **b** Top five protein classes and **c** top ten biological processes of significantly regulated genes independent of cell line. **d** Venn diagram showing differentially expressed genes for both cell lines after one single or multiple treatment cycles. **e** Two-tailed Pearson’s correlation of absolute gene expression fold change against toxicity (wild type) or viability (repeatedly exposed). **f** Log fold change of genes with a strong correlation in both SCC-25 and A431 cells, bar graphs show mean.

In order to identify genes correlating with cellular adaption towards recurrent oxidative stress conditions mediated by medical gas plasmas, whole-genome expression analysis was performed next. Therefore, RNA was isolated from wild-type and repeatedly exposed A431 and SCC-25 cells. Compared to untreated wild-type cells, gene expression was significantly altered after one or multiple treatment cycles in both cell lines (Fig. 4a). Differentially expressed genes with *P* < 0.05 and ≥fivefold change in expression levels were classified based on gene ontology (GO) analysis for protein class (Fig. 4b) and biological process (Fig. 4c). Strikingly, notable differences were not observed after one single or multiple treatment cycles, except for the expression of proteins related to immunity. In overlapping regions of the Venn diagram (Fig. 4d), 38 genes after one single (Table 2) and 10 genes after repeated exposure (Table 3) were identified to be differentially expressed in both A431 and SCC-25 cells. Overlapping genes were used for correlation analysis of absolute expression fold change against either toxicity (WT) or viability (RE) to evaluate genes that accounted for cellular sensitivity (WT) or resistance (RE). A strong correlation with the cellular sensitivity of wild-type cells was found for eight genes, including C5a anaphylatoxin chemotactic receptor 1 (C5AR1). After repeated exposure, altered expression of interleukin 1 receptor type 2 (IL1R2), dehydrogenase/reductase 9 (DHRS9), and kallikrein-related peptidase 13 (KLK13) showed the strongest correlation with resistance towards gas plasma treatment (Fig. 4e). Except for KLK13 and DHRS9, all significantly correlating genes were upregulated upon gas plasma treatment in both cell lines (Fig. 4f).

**Table 2:** Significantly correlating genes in SCC-25 and A431 squamous cell carcinoma cells after one single gas plasma treatment.

	SCC-25		A431	
Gene	*P* value	Log(FC)	*P* value	Log(FC)
*ACCSL*	3.95E-04	3.15	4.27E-04	2.44
*ATF3*	5.50E-03	4.00	1.12E-03	3.50
*AZGP1*	1.68E-02	2.43	3.57E-04	2.77
*C19orf18*	4.11E-05	2.82	4.47E-03	2.48
*C5AR1*	7.37E-04	2.48	1.08E-05	3.87
*CCZ1B*	1.36E-04	3.11	3.98E-05	2.51
*CHD2*	3.66E-05	3.44	2.06E-05	2.38
*COL20A1*	3.49E-03	2.45	1.08E-03	2.52
*DSC3*	4.48E-04	2.88	2.30E-04	2.52
*FAM214B*	4.31E-03	2.63	2.93E-04	2.62
*FCN1*	1.16E-03	3.11	1.18E-05	2.83
*GACAT1*	3.53E-04	2.59	4.29E-05	2.51
*GAL3ST1*	1.24E-03	2.72	1.31E-05	3.09
*GGN*	7.76E-05	2.51	9.58E-06	2.47
*GS1-24F4.2*	1.62E-03	2.33	2.09E-06	3.38
*HAAO*	2.58E-03	2.85	5.05E-06	2.67
*ID2*	4.08E-02	3.13	2.88E-03	2.35
*IL11*	5.77E-07	4.92	1.10E-05	3.63
*ILDR1*	3.34E-03	2.67	3.70E-06	2.37
*INHBE*	1.28E-02	3.53	1.71E-04	2.65
*IRAK2*	1.10E-03	2.62	2.38E-03	2.64
*L3MBTL2-AS1*	1.85E-02	2.35	4.90E-04	2.63
*MAP4K3-DT*	3.16E-04	2.49	1.09E-04	2.47
*MROH5*	1.72E-04	3.12	7.59E-05	2.35
*NUPR1*	3.67E-02	2.65	2.11E-06	2.95
*OLIG3*	6.07E-03	2.46	1.46E-02	2.36
*POMZP3*	2.47E-03	2.52	3.24E-05	2.33
*PRDM1*	8.18E-05	2.53	4.55E-06	2.76
*RAB6C-AS1*	7.66E-05	2.72	1.18E-05	2.55
*RUFY3*	1.13E-02	2.49	7.67E-05	2.91
*S100P*	1.41E-02	2.47	8.38E-05	2.61
*SLC6A9*	1.02E-02	2.42	2.57E-04	2.43
*TMEM200C*	4.13E-03	2.64	5.30E-04	2.82
*TMEM35A*	6.80E-03	2.80	4.24E-03	2.70
*TRARG1*	1.56E-03	2.52	1.73E-04	2.78
*TRIM15*	3.10E-04	2.83	2.43E-05	2.66
*ULBP1*	3.04E-04	3.08	2.83E-03	2.81
*ZNF807P*	2.09E-03	2.57	1.28E-05	2.63

The table is alphabetically sorted for genes showing increased or decreased log fold changes (FC) in mRNA levels and *P* values.

**Table 3:** Significantly correlating genes in SCC-25 and A431 squamous cell carcinoma cells after repetitive gas plasma treatment.

	SCC-25		A431	
Gene	*P* value	Log(FC)	*P* value	Log(FC)
*DHRS9*	2.12E-04	−2.61	1.15E-02	2.68
*EDN2*	6.16E-05	−2.77	2.10E-03	3.25
*H19*	1.16E-06	−4.03	4.61E-05	2.78
*H19*	4.79E-06	−3.37	9.51E-06	3.55
*IL1R2*	1.80E-03	2.78	6.37E-03	2.58
*KLK13*	1.94E-05	−2.87	3.88E-04	2.66
*LY6D*	4.27E-04	−3.34	1.32E-04	2.44
*PBX1*	6.23E-05	−2.44	1.91E-05	3.38
*RAET1E*	1.04E-04	−2.39	1.57E-04	2.36
*TNNT3*	3.64E-04	−3.38	1.80E-03	2.63

The table is alphabetically sorted for genes showing increased or decreased log fold changes (FC) in mRNA levels and *P* values.

### Repeatedly exposed cancer cells exhibit slow-cycling behaviour in vivo

**Fig. 5:**
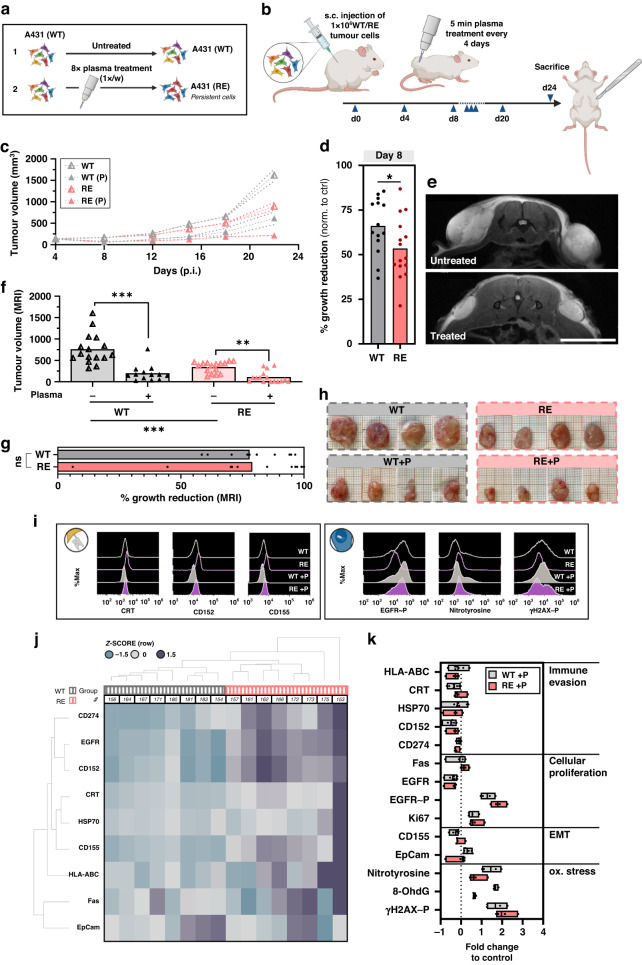
Repeatedly exposed cancer cells exhibit slow-cycling behaviour in vivo. **a** Experimental summary of generation of repeatedly exposed (RE) A431 cells for in vivo experiments. **b** Experimental procedure of animal experiments (*n* = 8, except WT + P (*n* = 7)). **c** Caliper measurements of tumour growth (*n* = 16, except WT + P (*n* = 14). **d** Growth reduction of WT and RE tumours in response to gas plasma treatment calculated from caliper measurements at day 8 (RE, *n* = 16; WT, *n* = 14), graph shows mean with individual data points, statistical analysis was performed using unpaired *t* test (one-tailed *P ≤ 0.05). **e** representative MRI images and **f** calculated tumour volume thereof (*n* = 16, except WT + P (*n* = 14)), bar graphs show mean and individual data points, statistical analysis was performed using one-way analysis of variance (ANOVA) (**P ≤ 0.01, ***P ≤ 0.001). **g** Growth reduction of WT and RE tumours in response to gas plasma treatment calculated from the area under the curve of MRI volume measurements (RE, *n* = 16; WT, *n* = 14), bar graphs show median and individual data points, statistical analysis was performed using unpaired *t* test (two-tailed). **h** Representative images of excised tumours. **i** Representative flow cytometry intensity histograms of calreticulin (CRT), CD152, CD155 (extracellular staining) and phosphorylated epithelial growth factor receptor (EGFR ~ P), nitrotyrosine, phosphorylated histone 2AX (yH2AX~P, intracellular staining). **j** WPGMA-weighted hierarchical clustering of z-scored baseline surface marker expression levels of untreated WT and RE tumours (*n* = 8). **k** Fold change of extra- (*n* = 7) and intracellular (*n* = 4) marker expression of gas plasma-exposed WT and RE tumours normalised to untreated controls, floating bars show minimum to maximum, with the mean indicated as a line. ns non-significant, WT wild type, RE repeated exposure, p plasma, ox stress oxidative stress, EMT epithelial–mesenchymal transition, UTR untreated.

Up to this point, the in vitro data underlined morphological, functional, and genetic alterations of A431 and SCC-25 cells after multiple gas plasma treatment cycles that were associated with a more resistant phenotype. In order to evaluate treatment responses of A431 that persisted after repeated exposure (RE) to gas plasma treatment compared to wild-type cells (Fig. 5a), a xenograft squamous cell carcinoma model was employed (Fig. 5b). Tumour-bearing mice were exposed to argon gas plasma every 4 days until day 24.

Gas plasma treatment diminished the tumour growth in mice engrafted with WT and RE cells compared to the respective controls (Fig. 5c) and a significantly lower growth reduction was observed in RE tumours until day 8 (Fig. 5d). Magnetic resonance imaging (Fig. 5e) revealed a markedly decelerated growth rate of untreated RE cells compared to WT cells and calculated tumour volumes confirmed a gas plasma-induced reduction in both WT and RE tumour burden (Fig. 5f). However, at the last day of the in vivo experiment, no significant difference in growth reduction was observed between both cell types (Fig. 5g). Excised tumours (Fig. 5h) were digested to create single-cell suspensions for flow cytometric analysis of intracellular or surface marker expression (Fig. 5i). WPGMA-weighted hierarchical clustering of z-scored surface marker expression in untreated WT and RE tumours showed notable differences at baseline levels with an overall upregulation for the majority of investigated targets revealing a non-immunogenic and pro-tumorigenic phenotype of RE tumours (Fig. 5j). Gas plasma treatment reduced the expression of immune-inhibitory CD152 and CD274 but also ICD-related calreticulin (CRT) in WT tumours. Likewise, the expression of epithelial growth factor receptor (EGFR) and proliferation marker Ki-67 was reduced. This was paralleled by increased expressions of epithelial cell adhesion molecule (EpCam) and oxidative stress-related markers (Supplementary Fig. S5a). Strikingly, fewer alterations were found in RE tumours after gas plasma treatment (Supplementary Fig. S5b). Compared to wild-type tumours, RE tumours displayed a notably lower increase in oxidative stress-related nitrotyrosine and 8-hydroxydesoxyguanosin (8-OhdG) but elevated levels of phosphorylated EGFR (EGFR ~ P) (Fig. 5k).

**Fig. 6:**
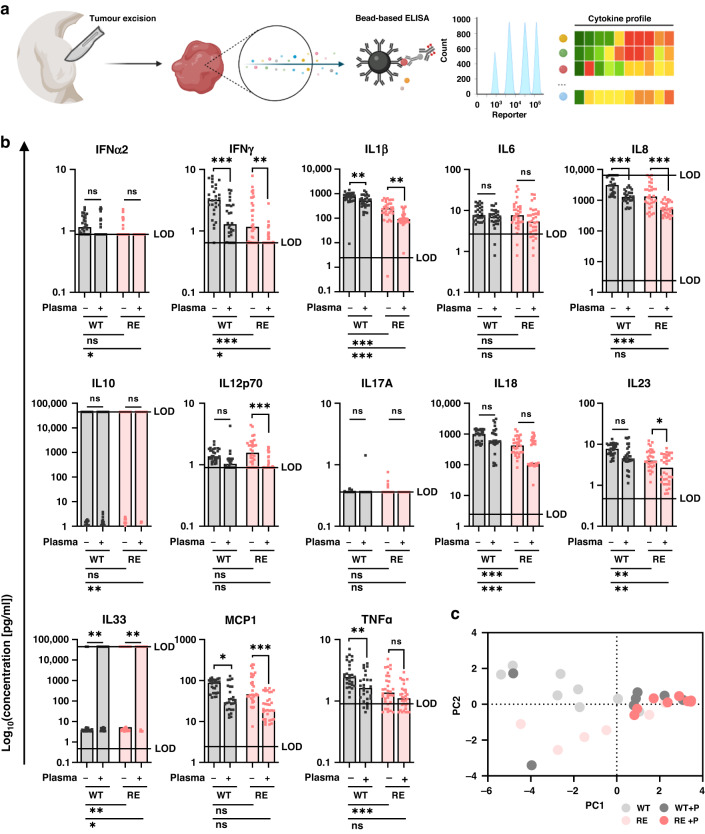
Gas plasma treatment modulates the cytokine profile of WT and RE tumours in vivo. **a** Schematic overview of the experimental procedure for assessment of cytokine profiles in excised tumours. **b** Absolute cytokine and chemokines levels, bar graphs show median with individual data points. Statistical analysis was performed using ordinary two-way analysis of variance (ANOVA) with Šídák’s post hoc testing (*P ≤ 0.05, **P ≤ 0.01, ***P ≤ 0.001). **c** Principal component analysis (PCA) calculated from z-scored chemokine and cytokine levels of all animals (dots represent individuals of groups). ns non-significant, LOD limit of detection, WT wild type, RE repeated exposure, p plasma.

Using a bead-based multianalyte assay, cytokine release was next evaluated in the interstitial fluid of excised and digested tumours (Fig. 6a). IL10 and IL33 concentrations were not reliably detectable in nearly all groups of our experiment since values exceeded the limit of detection (indicated as line). A significant decrease in levels of interferon γ (IFNγ), IL1β, interleukin 8 (IL8) and monocyte chemoattractant protein-1 (MCP1) was identified in both gas plasma-treated WT and RE tumours. By contrast, tumour necrosis actor α (TNFα) was decreased in the former, while levels of interleukin 12p70 (IL12p70) and interleukin 23 (IL23) were reduced in gas plasma-treated RE tumours. Overall, A431 RE tumours showed a diminished chemokine and cytokine profile being significantly lower for the majority of targets. (Fig. 6b). Principal component analysis calculated from z-scored cytokine levels underlined that WT and RE tumours showed similar responses to medical gas plasma across all animals (Fig. 6c).

## Discussion

Therapeutic resistance of cancers can cause failure of proven oncological approaches, leading to tumour relapse, metastasis, and high mortality rates. ROS-based approaches, including photodynamic therapy (PDT) and other emerging technologies such as medical gas plasmas, have been considered for targeting multidrug-resistant cancers [24]. Experimental evidence suggests that cancers evolve and adapt under recurrent oxidative stress conditions as well. Investigation of underlying mechanisms is urgently needed to increase knowledge on adaption processes and identify eligible targets to expand therapeutic opportunities for affected patients and their attending clinicians. Notwithstanding, cancer resistance can occur due to various mechanisms, including specific genetic or epigenetic changes and also the influence of the microenvironment in which the cell resides. Thus, tumour cell fate is governed by a complex matrix of contributing, interconnected factors that complicate the answer to this fundamental question.

In order to investigate the evolvement of therapeutic resistance towards gas plasma-derived ROS, we established a standardised model of repeated gas plasma treatment in two squamous cell carcinoma cell lines in vitro. Cells were exposed to medical gas plasma weekly in eight treatment cycles following downstream analysis of cellular and subcellular responses. Compared to their wild-type counterparts, A431 and SCC-25 cells were found to be more resistant after multiple treatment cycles. Similar observations have been reported for approaches that trigger endogenous ROS formation in cancer cells, including ionising radiation or various chemotherapeutic drugs [25–28]. Here, cancer cell adaption has been linked to the stemness and evolvement of slow-cycling cells under prolonged ROS stress. Slow-cycling cells are characterised by their quiescent nature and high resistance and are considered a significant cause for tumour relapse in oncology. G1/G0 arrest and altered expression of cell cycle-related genes are moreover associated with resistance towards hydrogen peroxide, one of the best characterised ROS in redox biology [29]. Although repeatedly exposed A431 cells showed slow-cycling behaviour in vivo, a baseline increase of cells arrested in G1 phase was not observed after multiple treatment cycles in vitro. By contrast, gas plasma treatment increased the proportion of repeatedly exposed A431 in G2 phase, indicative of cellular senescence. Cancer cells that exit the cell cycle after the S-phase are considered polyploid giant cancer cells (PGCCs), which avoid lethal damage by entering a non-dividing cell state, allowing them to adapt to a toxic environment and generate highly resistant clonal subpopulations after reinitiating proliferation [30–33]. Increased resistance and apoptosis evasion of head and neck cancer cells has been recently linked to G2/M phase arrest in vitro and patient-derived tissues [34, 35]. In our study, the evolution of PGCCs with a stem-like phenotype is also indicated by visual cell size enlargement in vitro [33].

Despite alterations in cell cycle distribution, squamous cell carcinoma cells displayed increased baseline ROS/RNS levels indicated by elevated tyrosine nitration and oxidised GSH (GSSG) after repeated exposure. Interestingly, this contrasts the general assumption that low intracellular GSH levels increase cellular susceptibility toward ROS-induced cytotoxicity [36]. However and in contrast to WT cells, further consumption of GSH could not be observed in RE cells, supporting the conjecture of an altered antioxidant defence able to maintain stable levels of intracellular ROS upon plasma treatment. This aligns with previous findings indicating that not basal GSH levels but differences in GSH metabolism are associated with cellular sensitivity toward plasma treatment [37]. A strengthened antioxidant defence and oxidative adaption were further outlined by increased intracellular levels of catalase and SOD1 observed in our study. An initial decrease of SOD1 expression 6 h after plasma treatment might indicate mitochondrial dysfunction upon acute oxidative stress in WT and RE cells [38]. Dynamics of oxidative stress adaption are influenced by exposure intervals and absolute levels of the respective oxidative agents. So far, most studies have focused on cellular adaptation induced by one acute stress, whereas physiologically or in therapeutic settings, cells are challenged by repeated or chronic oxidative stress events. Oxidative stress adaption involves a 24–30 h time period in which cells shift their gene expression patterns in response to an oxidative stressor and gradually de-adapt if the stressor is removed [39]. In a study performed by Pickering and colleagues, oxidative adaption after repeated exposure to H_2_O_2_ did only occur at sufficiently long time intervals and given that dose levels of repeated or chronic stress were lower than optimal for adaptation to an acute stressor [40]. In this study, translating to the clinical situation, cancer cells were exposed to a repetitive, not continuous or additive stressor, as ROS-enriched cell culture medium was removed 24 h after treatment. Due to the variety and high reactivity of radicals and ROS/RNS generated by gas plasmas monitoring their nonlinear dynamics is complex. The majority of ROS generated by gas plasmas have very short lifetimes, including hydroxyl radicals with a half-life in biological systems of about 1 ns, singlet oxygen and superoxide with a half-life of about 1–4 µs, peroxynitrite with a half-life of about 1 s, and hydroperoxyl radicals with half-lives of <10 s in aqueous solutions [41–43]. Thus, a prolonged exposure of cells towards the mentioned species is not expected. However, secondary reactions cause the formation of more stable species, mainly hydrogen peroxide in the case of an argon plasma jet [44]. In our study, 15 s plasma exposure, corresponding to the predetermined IC25 value, yielded deposition of 40 µM H_2_O_2_ without cells, remaining stable over the time course of 24 h. Interestingly, absolute levels of H_2_O_2_ were strikingly reduced in the presence of cells, indicating the consumption of species [45]. Similar observations were made after relative assessment of short-lived species, including hydroxyl radicals, peroxynitrite, hypochlorous acid, singlet oxygen and nitric oxygen.

ROS/RNS exhibit pleiotropic functions in various physiological signalling pathways and are crucial for cellular proliferation, migration, and angiogenesis [46, 47]. Due to specific biochemical alterations, elevated intracellular ROS levels have been reported in many malignancies, supporting largely all hallmarks of cancer in oxidative eustress conditions. Thus, low levels of endogenous ROS contribute to tumorigenesis [48] and are associated with resistant, stem-like phenotypes [49–51]. At the same time, excessive ROS exposure can elicit oxidative distress and lethal damage to tumour cells. Repeated exposure to the mixture of gas plasma-derived ROS might represent a constant selection pressure for stem-like cancer cells that exploit the hormetic principle of ROS [50, 52]. As cancer stem cells (CSCs) are considered a major cause of tumour recurrence and resistance establishment towards a broad spectrum of conventional chemotherapeutics [53] and radiation [54], the selection of CSCs or cancer cells with stem-like characteristics upon repeated plasma exposure could be a major cause for tumour recurrence and treatment failure after initial therapeutic response [55]. Hence, developing combinational treatment approaches targeting CSCs could be critical to improving tumour remission rates in clinical oncology [55] and, consequently, outcomes in applied plasma medicine in the future. This study showed a strong correlation between acquired plasma resistance and upregulation of immunosuppressive IL1R2, suggesting its potential role as a biomarker and target for novel therapeutic approaches. In addition, several anti-CSC therapies are under investigation, including the administration of all-*trans* retinoic acid to induce CSC terminal differentiation [56], inhibition of key CSC signalling pathways [57] or CSC ablation using antibody-drug conjugates such as CD33 in AML [58]. In addition, future studies should aim to optimise treatment regimes in terms of treatment length, intervals and frequency to maximise therapeutic responses while minimising side effects and the risk of resistance establishment.

Interestingly, RE cells maintained their resilient phenotype only until day 8 in vivo, following gradual de-adaption. Important to note, persistent RE cells evolved under exposure to long-lived ROS/RNS chemistries in vitro. Here, cells are surrounded by bulk liquids that cause quick deterioration of species generated in the gas phase [59–61]. In vivo, however, short-lived ROS/RNS chemistries play a major role and might outperform long-lived species concerning toxicity due to their high reactivity. Adaption of cells in vitro might have been insufficient in this regard. Marked differences were observed in baseline surface marker expression profiles in WT and RE tumours, underlining the evolvement of different phenotypes after multiple treatment cycles. RE tumours had elevated baseline levels of epithelial growth factor receptor (EGFR), which is highly overexpressed in various malignancies and associated with resistance and poor prognosis [62–64]. Upon ligand binding, its trans-auto-phosphorylation, enhanced in gas plasma-treated RE tumours, activates multiple signalling pathways contributing to tumorigenesis [65]. Notably, decreased expression of the cell adhesion molecule EpCam was not observed in WT or RE tumours. Its surface expression impairs epithelial–mesenchymal transition (EMT) [66], and low expression levels are related to metastasis and tumour progression [67, 68]. As a major limitation, in vivo experiments were carried out in immunodeficient mice to enable the engraftment of human cancer cells. This eventually masked the impact of immune escape in RE tumours, as expression of checkpoint molecules CD274 (PD-L1; programmed cell death ligand 1) and CD152 (CTLA-4; cytotoxic T-lymphocyte-associated protein 4) were found to be markedly increased at baseline levels. The binding of CTLA-4 to CD80/CD86 or PD-L1 to PD-1 suppresses T-cell activation, impairing tumour immune response and promoting a tolerogenic tumour microenvironment (TME) [69, 70].

TME remodelling is a remarkable tool for cancer cells, through which they take advantage of the bilateral interaction with stromal and immune cells and improve growth conditions. Besides alterations in immune-related surface marker expression, cancers actively shape the TME by releasing tumour-promoting cytokines [71]. In vivo, a reduction was observed for the majority of targets after gas plasma exposure in both WT and RE tumours with similar patterns as indicated by principal component analysis (PCA). Hereof, increased levels of IL1β, IL8, MCP1 and TNFα have been related to tumour progression and metastasis in HNSCC [72–74]. IL1β has been shown to promote carcinogenesis and drug resistance via modulating Snail (SNAl1) and E-cadherin expression [75, 76]. Elevated levels of IL8 have been linked to poor prognosis in patients suffering from HNSCC by activating signalling downstream RAS/MAPK and STAT3 (signal transducer and activator of transcription 3) [77, 78], and release of MCP1 supports the recruitment of monocytes and tumour-associated macrophages (TAM) [74, 79]. TNFα can stimulate a Th1-type immune response [80–82], but its presence in the TME of HNSCC tumours has been shown to increase angiogenesis, invasiveness, and metastasis [72, 83]. In vitro, increased levels of pro-angiogenetic mediators, including platelet-derived growth factor-AA (PDGF-AA) and VEGF, were found in repeatedly exposed cells [84, 85].

Whole-genome expression analysis revealed a strong correlation between acquired resistance and stem cell characteristics and increased expression of IL1R2, an immunosuppressive regulator overexpressed in various tumour entities and linked to poor prognosis [86]. IL1R2 has been shown to enhance stem cell self-renewal of breast cancer cells through deubiquitination of BMI1 [87], increased angiogenesis, and proliferation by interaction with the transcriptional factor c-Fos in colorectal cancer [88], and elevated ZEB2 expression in prostate cancer leading to augmented migration and invasiveness [89]. In this light, IL1R2 expression could serve as a biomarker to estimate the therapeutic success of medical gas plasma and other ROS-based approaches in HNSCC. Moreover, targeting IL1R2 using neutralising antagonists has already been shown to inhibit cancer cell growth, invasion, and chemoresistance in vitro [87], and might be considered an approach to overcome resistance in gas plasma oncology in the future.

We have shown that repeated exposure to gas plasma-derived ROS/RNS can trigger a resilient phenotype in squamous cell carcinoma cells with stem-like characteristics. Altered expression of EGFR or release of VEGF could serve as eligible targets to overcome gas plasma oncology in multimodal approaches with approved inhibitors already applied in oncological regimes. Likewise, therapeutically targeting IL1R2 is promising, but underlying signalling pathways remain to be elucidated.

## Conclusion

Repetitive gas plasma exposure elicits a more insensitive cancer cell phenotype associated with increased cancer stem cell properties like an augmented G2-phase arrest, a higher intracellular basal level of ROS, and cell size enlargement. Further, enhanced secretion of pro-angiogenetic molecules in vitro and raised expression of anti-immunogenic surface markers in vivo unveiled a more tumorigenic and less inflammatory phenotype of persistent cells. IL1R2 was associated with the established unresponsiveness rendering this receptor a potential target for overcoming gas plasma-induced resistance and developing novel therapeutic approaches.

## Supplementary information

Supplemental Figures

## Data Availability

Data of this manuscript are available upon reasonable request.
