# Supplementary material for: Chronic oxidative stress adaptation in head and neck cancer cells generates slow-cyclers with decreased tumour growth in vivo
Source: Br J Cancer. 2023 Jul 17;129(5):869–83. doi: 10.1038/s41416-023-02343-6 (PMC10449771; doi:10.1038/s41416-023-02343-6)
Supplement: Supplementary file 1 — Supplemental Figures [file 41416_2023_2343_MOESM1_ESM.pdf]

Chronic oxidative stress adaptation in head and neck cancer cells generates slow-cyclers with decreased tumor growth in vivo

Julia Berner[1,2], Lea Miebach[2,3], Marcel Kordt[4], Christian Seebauer[1], Anke Schmidt[2], Michael Lalk[5], Brigitte Vollmar[4], Hans-Robert Metelmann[1], *Sander Bekeschus[2,6]

1 Department of Oral, Maxillofacial, and Plastic Surgery, Greifswald University Medical Center, Ferdinand-Sauerbruch-Str, 17475 Greifswald, Germany

2 ZIK *plasmatis*, Leibniz Institute for Plasma Science and Technology (INP), Felix-Hausdorff-Str. 2, 17489 Greifswald, Germany

3 Department of General, Visceral, Thoracic, and Vascular Surgery, Greifswald University Medical Center, Ferdinand-Sauerbruch-Str, 17475 Greifswald, Germany

4 Rudolf-Zenker-Institute of Experimental Surgery, Rostock University Medical Center, Schillingallee 69a, 18057 Rostock, Germany

5 Institute for Biochemistry, University of Greifswald, Felix-Hausdorff-Str. 4, 17489 Greifswald, Germany

6 Clinic and Policlinic for Dermatology and Venerology, Rostock University Medical Center, Strempelstr. 13, 18057 Rostock, Germany

*correspondence: sander.bekeschus@inp-greifswald.de

Supplemental Figures

Figure S1

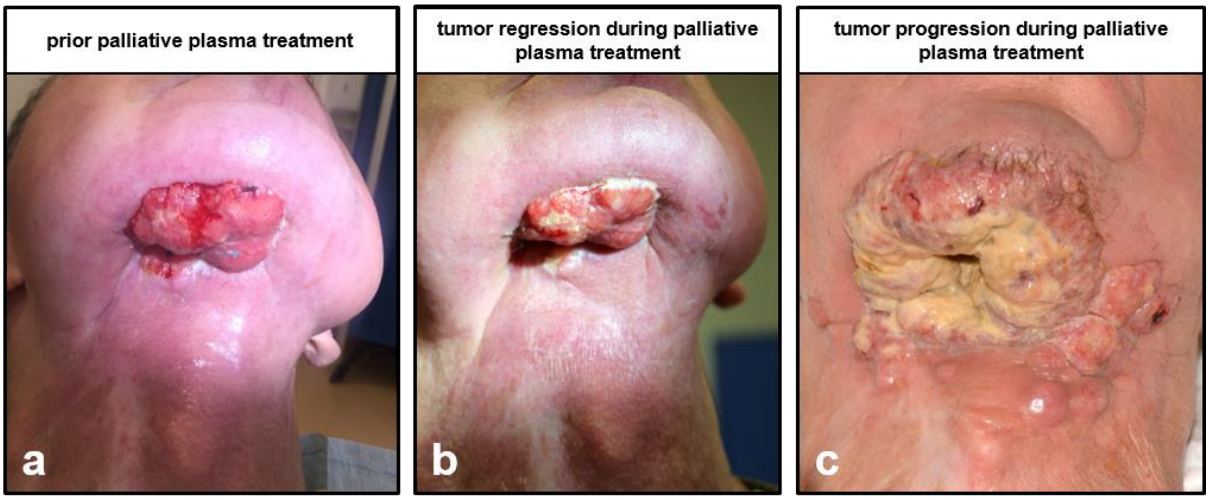

Figure S1. Acquired resistance to medical gas plasma therapy impairs tumor remission in a 54-year-old HNSCC patient. (**a**) extraoral exulcerated and bacterially superinfected tumor area prior to palliative gas plasma treatment; (**b**) partial tumor remission after repeated gas plasma application; (**c**) progressive disease after eight months of gas plasma therapy.

Figure S2

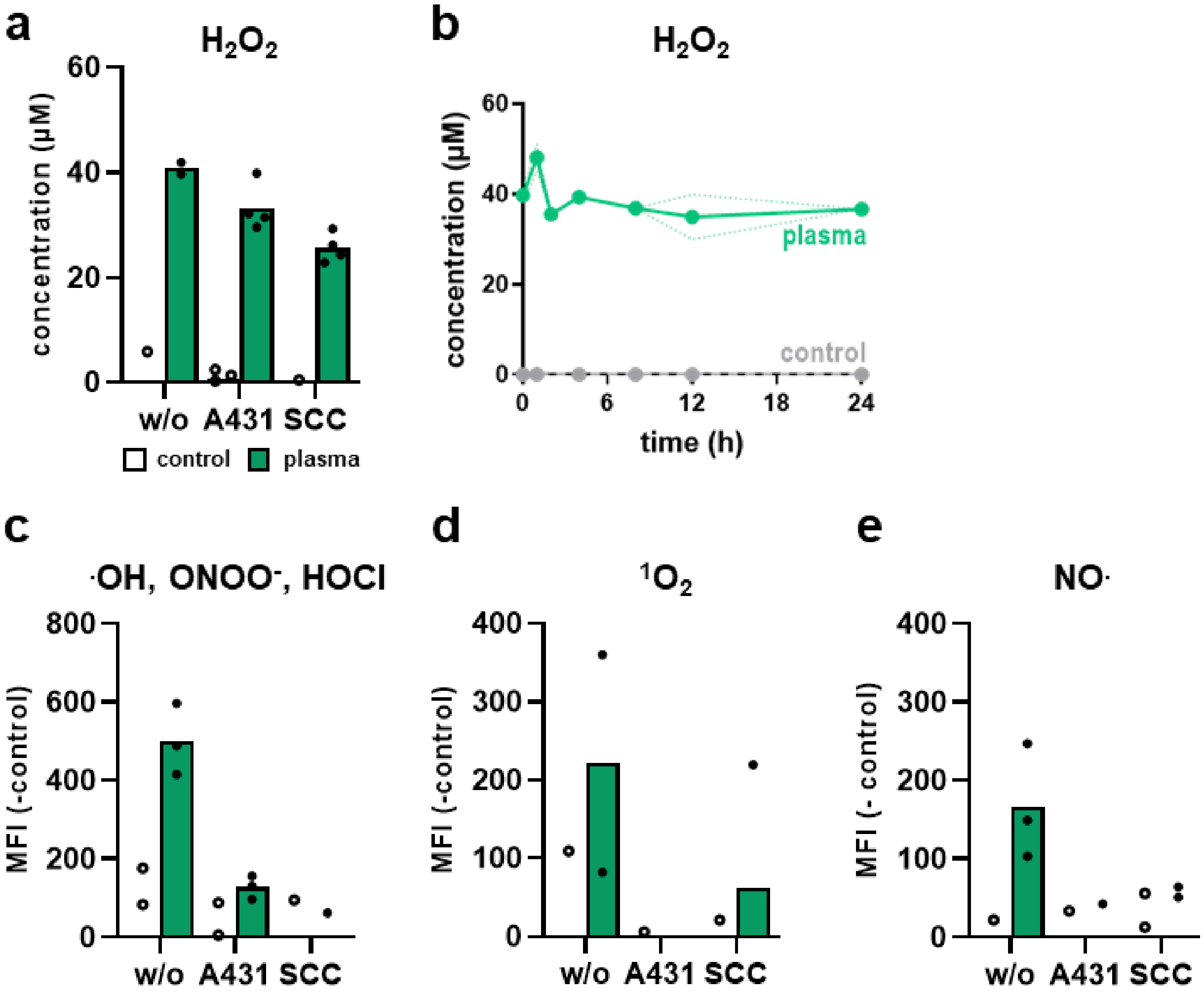

Figure S2. Reactive species profiling of plasma treatment conditions. (**a**) Quantification of hydrogen peroxide (H_2O_2) levels in the absence or presence of wildtype A431 and SCC-25 cells (n=4); (**b**) assessment of H_2O_2 stability over a time course of 24h (n=4); (**c-e**) deposition of short-lived reactive oxygen and nitrogen species in the absence or presence of wildtype A431 and SCC-25 cells using redox-sensitive probes indicative of hydroxyl radicals (·OH), peroxynitrite ($ONOO^-$), hypochlorous acid (HOCl; **b;** n=4), singlet oxygen ($^1O_2$; **c;** n=3), and nitric oxide (NO; **d**; n=3·). Bar graphs show mean with individual data points.

Figure S3

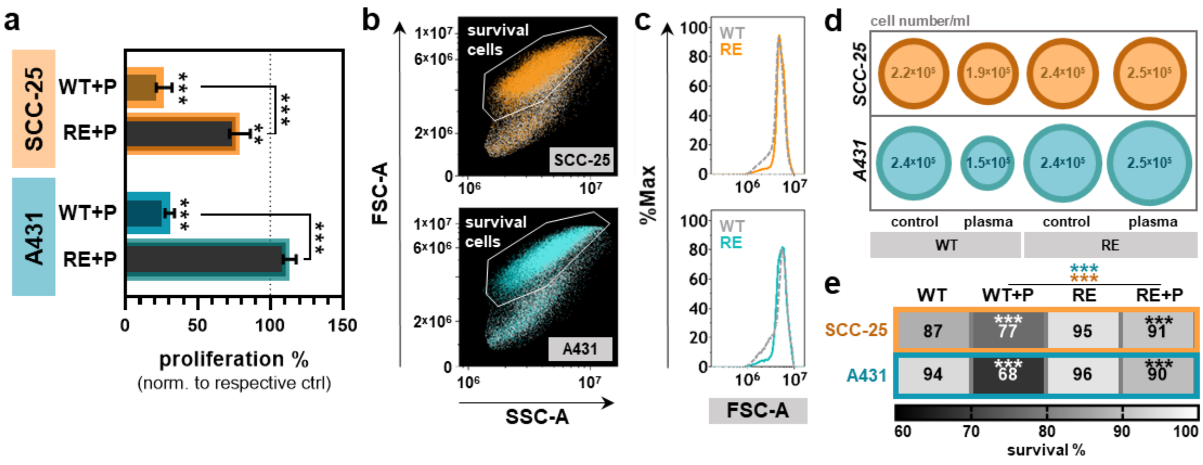

Figure S3. Validation of acquired resistance upon repeated plasma treatment. (**a**) proliferation of plasma-treated WT (n=7) and RE (n=6) cells 24h after exposure, bar graph shows mean ± standard error of the mean (SEM), statistical analysis was performed using ordinary two-way analysis of variance (ANOVA) with Šídák's post-hoc testing (**p≤0.01, ***p≤0.001); (**b**) representative flow cytometry dot plots and histograms (**c**) of plasma-exposed WT (grey) and RE (colored) cells 24h post-treatment; (**d**) calculated cell number/ml of control and treatment groups; (**e**) quantification of survival SCC-25 and A431 cells after one single or multiple treatment cycles compared to untreated controls (n=8), heat map shows mean, statistical analysis was performed using two-way analysis of variance (ANOVA) with Šídák's post-hoc testing (***p ≤ 0.001).

Figure S4

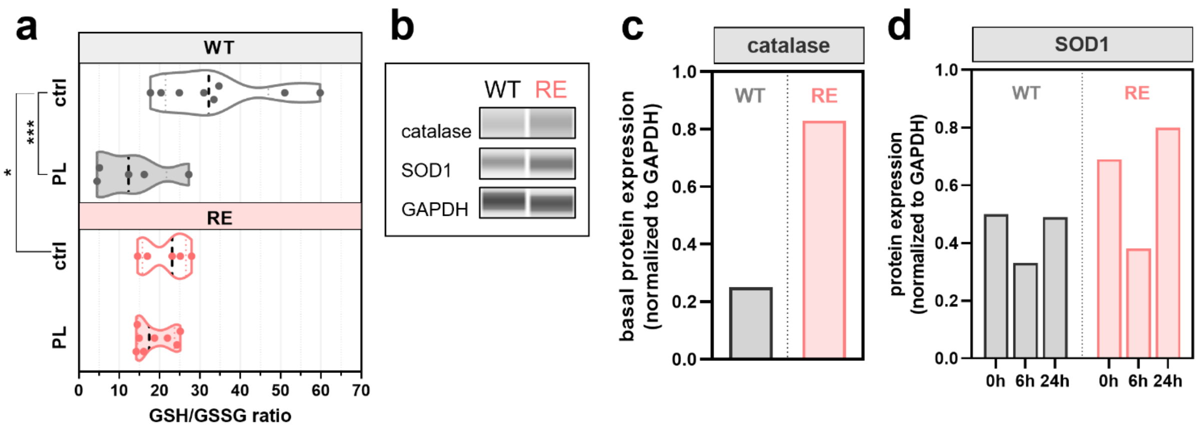

Figure S4. Oxidative adaption upon repeated plasma treatment. (**a**) GSH/GSSG ratio in control and treated wildtype or repeatedly exposed A431 cells (n=3), violin plots show mean (indicated as stacked line), quartiles (indicated as dotted line) and individual data points; (**b**) representative Western blot images of SOD1 and catalase expression in wildtype and repeatedly exposed A431 cells; (**c**) quantification of catalase baseline expression in wildtype and repeatedly exposed A431 cells, bar graphs show mean, statistical analysis was performed using ordinary one-way analysis of variance (ANOVA) (*$p \leq 0.05$, ***$p \leq 0.001$); (**d**) quantification of SOD1 expression in wildtype and repeatedly exposed A431 cells before as well as 6 h and 24h after gas plasma exposure, bar graphs show mean. WT = wildtype. RE = repeated exposure. PL = plasma.

56 *Figure S5*

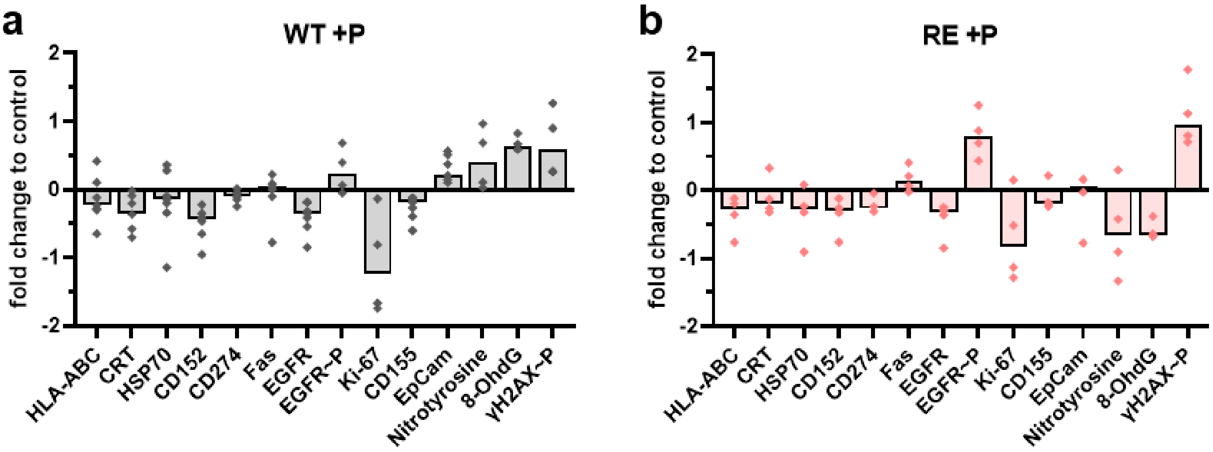

57

58 **Figure S5.** (**a**) *In vivo* gas plasma treatment alters the expression of intra- (n=4) and extracellular (n=7) markers
59 for immune evasion, cellular proliferation, EMT, and oxidative stress in WT and (**b**) RE tumors. ns = non-significant.
